# Arthropods communities in organic orchards and adjacent hedgerows on Terceira Island (Azores, Portugal): a dataset supporting biological control research

**DOI:** 10.3897/BDJ.14.e210298

**Published:** 2026-10-27

**Authors:** Sophie Wallon, António Domingues, Alejandra Ros-Prieto, Sébastien Lhoumeau, David H. Lopes, Paulo A. V. Borges

**Affiliations:** 1 University of Azores, CE3C—Centre for Ecology, Evolution and Environmental Changes, Azorean Biodiversity Group, CHANGE —Global Change and Sustainability Institute, School of Agricultural and Environmental Sciences, Rua Capitão João d’Ávila, Pico da Urze, 9700-042 Angra do Heroísmo, Azores, Portugal, Angra do Heroísmo, Azores, Portugal University of Azores, CE3C—Centre for Ecology, Evolution and Environmental Changes, Azorean Biodiversity Group, CHANGE —Global Change and Sustainability Institute, School of Agricultural and Environmental Sciences, Rua Capitão João d’Ávila, Pico da Urze, 9700-042 Angra do Heroísmo, Azores, Portugal Angra do Heroísmo, Azores Portugal; 2 Serviços de Desenvolvimento Agrário da Terceira, Angra do Heroismo, Azres, Portugal Serviços de Desenvolvimento Agrário da Terceira Angra do Heroismo, Azres Portugal; 3 IUCN SSC Atlantic Islands Invertebrate Specialist Group, Angra do Heroísmo, Azores, Portugal IUCN SSC Atlantic Islands Invertebrate Specialist Group Angra do Heroísmo, Azores Portugal; 4 IUCN SSC Monitoring Specialist Group, Angra do Heroísmo, Azores, Portugal IUCN SSC Monitoring Specialist Group Angra do Heroísmo, Azores Portugal

**Keywords:** beneficial arthropods, fruit production system, live hedge, Nature-based Solutions

## Abstract

**Background:**

Organic orchards and their associated hedgerows can function as important ecological infrastructures in agricultural landscapes by providing shelter, prey resources, floral resources and refuge sites for arthropods, including predators and parasitoids involved in biological control. These habitats may support diverse arthropod communities and contribute to the suppression of pest populations below economically damaging levels. Characterising arthropod assemblages is, therefore, important for understanding the biodiversity associated with agricultural systems and for supporting future research on biological control.

Such information is particularly relevant on oceanic islands, where geographical isolation, high levels of endemism and ongoing introductions of non-native species shape community composition and ecosystem functioning. Furthermore, island agricultural landscapes often comprise relatively small and fragmented habitat patches, making semi-natural elements, such as hedgerows, particularly important for maintaining biodiversity and supporting ecosystem services. Biodiversity-friendly agricultural practices may, therefore, contribute simultaneously to biodiversity conservation, ecosystem-service provision and the early detection of potentially invasive species.

Under the scope of the BIODIVERSA+ COMCHA project, arthropod assemblages were sampled in six organically managed mixed orchards and adjacent hedgerows on Terceira Island, Azores, Portugal, generating a dataset that enables future comparisons of community composition and diversity between these habitat types. Sampling was conducted in September 2025 and May 2026 using standardised methods, including SLAM (Sea, Land and Air Malaise) traps, nocturnal active aerial searches, nocturnal foliage beating and diurnal foliage beating. The resulting Darwin Core Archive is composed of Event and Occurrence tables and provides baseline information on arthropod diversity in island agroecosystems, constituting a valuable resource for future biodiversity monitoring and biological control research.

**New information:**

We provide an inventory of arthropods collected in EU-certified organic orchards and surrounding hedgerows on Terceira Island, based on 46 sampling events in September 2025 and 36 sampling events in May 2026 (82 sampling events in total), distributed across six localities and two habitat types: Mixed Orchards and Mixed Hedges.

During September 2025 and May 2026, a total of 15,739 specimens were recorded in 1,863 occurrences records, representing four arthropod classes, 18 orders and 86 families.

Of these specimens, 13,854 were identified to species or subspecies level, corresponding to 196 taxa. Overall, the dataset includes 299 morphospecies or taxonomic units.

Amongst these specimens, 10,596 specimens were recorded in 1,300 occurrence records in September 2025, while 5,143 specimens were recorded in 563 occurrence records in May 2026. The identified species/subspecies include 11 endemic taxa, 57 native non-endemic taxa, 104 introduced taxa and 24 taxa of uncertain origin. In terms of abundance, native non-endemic taxa were dominant, followed by introduced, endemic and uncertain colonization taxa. This dataset includes four species that are new records for Terceira Island: *Dipoena melanogaster* (C.L. Koch, 1837) (Araneae, Theridiidae), *Textrix pinicola* Simon, 1875 (Araneae, Agelenidae), *Stenocaecilius caboverdensis* (Meinander, 1966) (Psocodea, Caeciliusidae) and *Euconnus campestris campestris* (L.W. Schaufuss, 1866) (Coleoptera, Staphylinidae). These records expand current knowledge of the arthropod fauna of Terceira Island and contribute to a more comprehensive understanding of the biodiversity associated with organic orchards and adjacent hedgerows. Such baseline information is valuable for future studies investigating arthropod-mediated ecosystem services, including conservation biological control.

The dataset provides a first standardised baseline for comparing arthropod assemblages between orchard interiors and hedgerows in organic fruit-production systems on Terceira Island, supporting future assessments of biological control potential, habitat management, biosecurity surveillance and Nature-based Solutions in insular agroecosystems.

## Introduction

Arthropods are a major component of agricultural ecosystems and play important roles in ecological processes that influence crop production and agroecosystem functioning (Verma et al. 2023). Many taxa contribute to ecosystem services, such as biological control, pollination and organic matter decomposition, whilst others may act as pests or contribute to ecosystem disservices. Amongst the beneficial taxa, natural enemies, including predators and parasitoids, are particularly important because they can suppress pest populations and, thereby, support agricultural productivity (Losey and Vaughan 2006, Alford 2019). Amongst them, predatory and parasitoid species, such as spiders, ground beetles and parasitic wasps, play a particularly important role in regulating populations of herbivorous insects and other potential crop pests. These organisms provide a key ecosystem service known as natural biological control, which can reduce pest outbreaks and decrease reliance on chemical pesticides (Gerlach et al. 2013). Indeed, beneficial arthropods allow for a reduction in the number of phytosanitary treatments, in some cases even eliminating them entirely (Landis et al. 2005, Jarpla et al. 2024). Furthermore, in some cases, phytosanitary treatments are ineffective and pest management can only be successfully achieved through biological control, as illustrated by the chestnut gall wasp, *Dryocosmus kuriphilus*, which is controlled by the parasitoid *Torymus sinensis* (Santos et al. 2025). Nature-based solutions and ecosystem-based approaches should be prioritised in agricultural landscapes to enhance biodiversity and promote sustainable pest management.

Maintaining populations of natural enemies is widely regarded as an important component of sustainable agriculture and integrated pest management. Whilst numerous studies have reported positive relationships between the richness and abundance of natural enemies and pest suppression, the strength and direction of these relationships may vary amongst systems and depend on ecological context, species composition and community structure (Letourneau et al. 2009, Griffin et al. 2013, Dainese et al. 2019). Consequently, documenting arthropod diversity and community composition remains an important step towards understanding ecological interactions and ecosystem-service provision in agricultural landscapes. However, agricultural intensification and landscape simplification have often reduced the diversity and availability of habitats within agroecosystems (Gebhardt et al. 2023) and often create conditions that are unfavourable for the survival and reproduction of beneficial organisms. Therefore, managing the environment to maintain and strengthen populations of these natural enemies is becoming an increasingly important approach in agricultural pest management (Landis et al. 2000, Altieri and Nicholls 2020, Boinot et al. 2023, Ferrante et al. 2024). As a result, increasing attention has been given to the role of ecological infrastructures in supporting biodiversity within agricultural landscapes. European policies, including the Nature Restoration Regulation (EU 2024/1991) and the Common Agricultural Policy (EU 2021/2115), promote the introduction of high-diversity landscape features in arable land and intensively used grasslands, such as buffer strips, field margins with native flowers, hedgerows, trees, small woodlands, terrace walls, ponds, habitat corridors and stepping stones. The presence of natural vegetation around the production area is crucial to increase beneficial arthropods diversity (Galloway et al. 2021). Indeed, hedgerows and other semi-natural linear habitats may act as ecological reservoirs for many arthropod species, including predators and parasitoids that contribute to biological control (Ricci et al. 2009, Précigout and Robert 2022). To assess both the presence of these organisms and the success of management strategies, it is essential to obtain robust and reliable datasets.

In this context, oceanic islands represent unique systems in which to study biodiversity patterns in agricultural landscapes. Due to their geographical isolation, island ecosystems often harbour distinctive assemblages of endemic, native and introduced species and may be particularly susceptible to biological invasions. Furthermore, agricultural landscapes on islands frequently consist of relatively small and fragmented habitat patches, making semi-natural elements, such as hedgerows, potentially important reservoirs of biodiversity and connectors between habitats. These features may provide shelter, refuge, alternative food resources and breeding sites for a wide range of arthropods, including predators and parasitoids involved in biological control. The Azores Archipelago, located in the North Atlantic Ocean, hosts a mixture of endemic, native and introduced arthropod species (Borges et al. 2022), making it an appropriate system for investigating arthropod diversity associated with agricultural habitats and their adjacent semi-natural elements.

Across the Archipelago, agriculture is a major land use and an important component of the regional economy. Approximately 48.82% of the Azorean territory is occupied by agricultural land, with pastures alone accounting for 39.63% of the regional surface (de Almeida et al. 2020). Agricultural landscapes include permanent and semi-natural pastures, orchards and other cultivated habitats, forming a heterogeneous mosaic (Marcelino et al. 2021, Sieber et al. 2021). Organic farming has also expanded markedly, with the combined area under organic production and conversion increasing from 1,277.57 ha in 2020 to 4,547.57 ha in 2022 (SRAC 2024). As the effects of organic farming on biodiversity vary amongst taxonomic groups and landscape contexts (Bengtsson et al. 2005, Huang et al. 2022), documenting the diversity and distribution of arthropods, associated with Azorean organic agricultural systems, provides an essential baseline for investigating the ecological processes that underpin natural pest regulation and other ecosystem functions (Bengtsson et al. 2005, Letourneau et al. 2009). This data paper presents a dataset of arthropods collected in organic orchards and associated hedgerows on Terceira Island (Azores, Portugal). The dataset includes records from multiple arthropod groups, with particular emphasis on predatory taxa that constitute important components of natural-enemy assemblages and may contribute to biological pest control (Landis et al. 2000, Letourneau et al. 2009).

## General description

### Purpose

Nature-based Solutions (NbS) and ecosystem-based approaches should be prioritised in agricultural sytems to enhance biodiversity and promote sustainable pest management. This project aim to provide baseline data on arthropod species richness and abundance in organic orchards and their surrounding hedgerows on Terceira Island (Azores). These data will allow the assessment of the diversity of beneficial arthropods in hedgerows compared to the orchard itself.

### Additional information

In addition to a permanent agroforest monitoring plot at Vale da Fonte (Pico Island, Azores) (Lago et al. 2026), we established permanent agroforest monitoring plots at six biodiversity-friendly production orchard systems sites on Terceira Island to generate baseline data on terrestrial arthropods communities and support future research on biodiversity and ecosystem services in agricultural landscapes.

## Project description

### Title

Beneficial arthropods in organic orchards and hedgerows on Terceira Island (Azores, Portugal) under the scope of the COMCHA project.

### Personnel

The project was conceived by Paulo A.V. Borges and António B. Domingues.

Fieldwork: António B. Domingues and Paulo A.V. Borges.

Parataxonomists: António B. Domingues, Alejandra Ros-Prieto and Sophie Wallon.

Taxonomists: Paulo A.V. Borges and Luís Carlos Crespo.

Database management: Sébastien Lhoumeau, Sophie Wallon and Paulo A.V. Borges.

Darwin Core Database management: Sébastien Lhoumeau, Sophie Wallon and Paulo A.V. Borges.

### Study area description

The study took place in the Azorean Archipelago (North Atlantic Ocean), specifically on Terceira Island (38.712925, -27.234912). The Azores, of volcanic origin, experience a temperate oceanic climate, with mild temperatures year-round at lower elevations and generally high levels of precipitation. Sampling was carried out in organic orchard and hedgerow habitats distributed across the Island between 71 and 197 m a.s.l.

### Design description

The main objective of this study was to characterise the diversity and distribution of arthropods, with particular emphasis on taxa associated with biological control, in orchards managed under organic production on Terceira Island, comparing species composition and abundance between orchard interiors and adjacent hedgerows. Six orchards certified under the Organic Production System on Terceira Island were sampled via SLAM traps (Sea, Land and Air Malaise), Active Aerial Search (AAS) and Foliage Beating.

### Funding

Fieldwork was financed by the project EU BIODIVERSA + COMCHA Community-based change: local and traditional knowledge(s) in NbS, through a Grant from Fundo Regional da Ciência e Tecnologia (FRCT). Open data availability for the general public will be available through funding from AZORES BIOPORTAL was obtained from the project “Upgrading the Azorean Biodiversity Portal Infrastructure (AZORES BIOPORTAL- PORBIOTA) to Boost Biodiversity Research, Management and Education -PORBIOTA” (DRCID, ACORES2030-FEDER-03420600), co-financed by ERDF (European Regional Development Fund), through the Direção Regional do Planeamento e Fundos Estruturais, under the Açores 2030 Programme, at 85% and by the Autonomous Region of the Azores (RAA) at 15% through the Direção Regional da Ciência, Inovação e Desenvolvimento, via the PROSCIENTIA Incentive System, with reference M1.1.C/COFUND AÇORES 2030/2026/001 - PORBIOTA.

## Sampling methods

### Study extent

**Figure 1.**
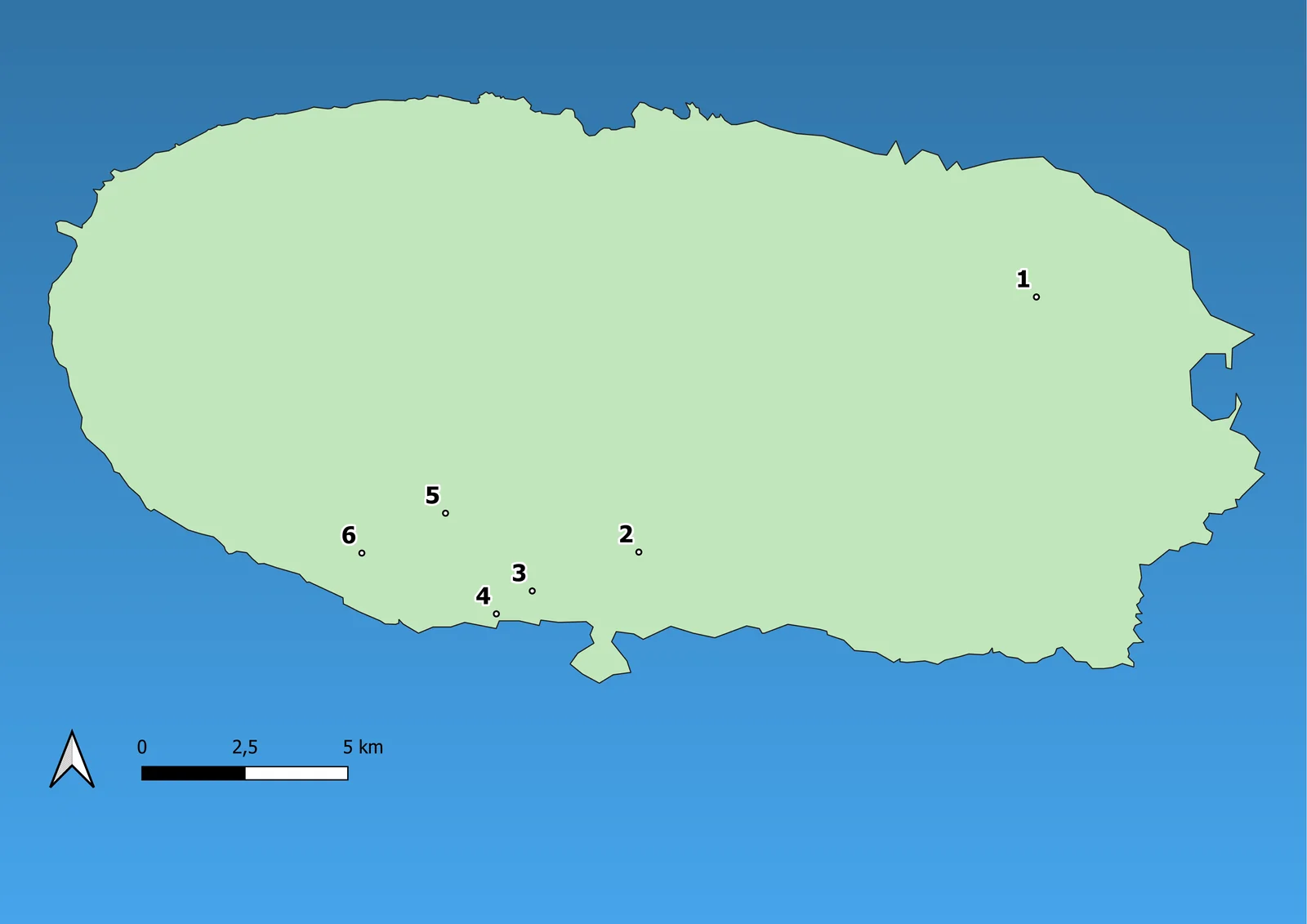
Localisation of the sampled orchards on Terceira Island. 1: Fontinhas; 2: Conceição; 3: São Pedro; 4: São Mateus; 5: Terra-Chã; 6: São Bartolomeu.

For the creation of this dataset, six orchards certified under the Organic Production System on Terceira Island were sampled (Fig. 1). All orchards are mixed, with five managed under the traditional Azorean system — characterised by the presence of hedgerows along plot borders and within the orchards, which provide protection against the strong winds common in this region — and one managed under a polyculture system (including fruit, aromatic and vegetable crops), with the dominant component being fruit trees and also surrounded by hedgerow. All hedgerows were mostly composed of banksia (*Banksia integrifolia* L.), pittosporum (*Pittosporum undulatum* Vent.) and faya (*Morella faya* (Aiton) Wilbur).

The geographic location of the six sampled orchard systems is summarised in Table 1. Five traditional orchards were respectively located in the parishes of São Mateus, Terra-Chã, São Pedro, Conceição and São Bartolomeu, whereas the polyculture system was located in the parish of Fontinhas. In each location, sampling was done in the middle of the orchard (Mixed Orchard) and along its edges (Mixed Hedges).

The composition and structure of each sampled orchard are detailed below:

São Mateus: The orchard is characterised by a polyculture system dominated by banana (*Musa acuminata* Colla) and coffee (*Coffea* sp.), with additional fruit trees along the margins, including avocado (*Persea americana* Mill.), Surinam cherry (*Eugenia uniflora*), mango (*Mangifera indica* L.) and papaya (*Carica papaya* L.).

Terra-Chã: This orchard is primarily composed of custard apple (*Annona cherimola* Miller) and avocado (*Persea americana* Mill.), with avocado trees present only in part of the cultivated area.

São Pedro: The orchard is dominated by citrus species, including orange trees (*Citrus sinensis* (L.) Osbeck) and mandarin (*Citrus clementina* hort. ex Tanaka), as well as coffee (*Coffea* sp.). Scattered individuals of loquat (*Eriobotrya japonica* (Thunb.) Lindl.), guava (*Psidium guajava* L.), avocado (*Persea americana* Mill.), custard apple (*Annona cherimola* Miller) and strawberry guava (*Psidium cattleyanum* Sabine) are also present.

Conceição: This orchard includes a mixture of cultivated species, such as blueberry (*Vaccinium* sp.), chestnut (*Castanea sativa* Mill.), feijoa (*Acca sellowiana* (O.Berg)), guava (*Psidium guajava* L.) and orange trees (*Citrus sinensis* (L.) Osbeck).

São Bartolomeu: The orchard is composed of citrus species (orange and mandarin), chestnut (*Castanea sativa* Mill.), coffee (*Coffea* sp.), custard apple (*Annona cherimola* Miller), loquat (*Eriobotrya japonica* (Thunb.) Lindl.) and plum (*Prunus domestica* L.).

Fontinhas: This site represents a diverse polyculture system including orange trees (*Citrus sinensis* (L.) Osbeck), coffee (*Coffea* sp.), chestnut (*Castanea sativa* Mill.), banana (*Musa acuminata* Colla), sapote (*Pouteria sapota* (Jacq.) H. E. Moore & Stearn), persimmon (Diospyros kaki L.f.), plum (*Prunus domestica* L.), custard apple (*Annona cherimola* Miller) and avocado (*Persea americana* Mill.).

Two SLAM (Sea, Land, Air Malaise) traps (see protocol details in Borges (2025)) were set in each location (one trap in the hedges and one in the orchard centre) during the month of September 2025 resulting in 12 samples.

For the Active Aerial Search (AAS) and foliage beating, the same protocols have been applied in all locations (hedges and orchard centre), in September 2025 and May 2026:

- 30 minutes of Active Aerial Search (AAS) at night;

- 30 minutes of foliage beating at night;

- 1 hour of foliage beating during the day.

A total of 82 sampling events were expected under this protocol (48 in September 2025 and 36 in May 2026). However, in September 2025, two foliage beatings at night could not be carried out, meaning the final number of events, from all methods combined, was 46: 12 AAS, 10 nocturnal foliage beatings and 12 diurnal foliage beatings and 12 SLAM traps. The samplings carried out in May 2026 are complete, with 36 sampling events (12 AAS, 12 nocturnal foliage beatings and 12 diurnal foliage beatings).

### Quality control

Arthropod specimens collected were transported to the laboratory, where individuals were sorted and identified to species level using a stereomicroscope and specific taxonomic literature. Specimens were initially sorted by parataxonomist António Domingues and subsequently identified by Paulo A. V. Borges. Juvenile individuals were included in the dataset, as the low species diversity in the Azores allows for reliable identification.

### Step description

A reference collection of Azorean arthropods, housed at the Dalberto Teixeira Pombo Insect Collection (University of the Azores), was used to help specimen identification. The taxonomic nomenclature and establishment status of the species follows the most recent checklist of Azorean arthropods (Borges et al. 2022).

## Geographic coverage

### Description

Terceira Island, Azores, Portugal.

### Coordinates

38.658 and 38.746 Latitude; -27.292 and -27.104 Longitude.

## Taxonomic coverage

### Description

The following classes and orders of arthropods are covered:

**Arachnida**: Araneae, Opiliones, Pseudoscorpiones; **Chilopoda**: Lithobiomorpha, Scutigeromorpha; **Diplopoda**: Julida; **Insecta**: Archaeognatha, Blattodea, Coleoptera, Dermaptera, Hemiptera, Hymenoptera, Lepidoptera, Neuroptera, Orthoptera, Phasmida, Psocodea, Thysanoptera.

### Taxa included

**Table:** 

Rank	Scientific Name	Common Name
class	Arachnida	Arachnids
order	Araneae	Spiders
order	Pseudoscorpiones	False scorpions
class	Chilopoda	Centipedes
order	Lithobiomorpha	Centipedes
order	Scutigeromorpha	Centipedes
class	Diplopoda	Millipedes
order	Julida	Millipedes
class	Insecta	Insects
order	Archaeognatha	Bristletails
order	Blattodea	Cockroaches
order	Coleoptera	Beetles
order	Dermaptera	Earwigs
order	Hemiptera	Bugs
order	Hymenoptera	Ants
order	Lepidoptera	Butterflies, Moths
order	Neuroptera	Lacewings
order	Orthoptera	Crickets, Grasshoppers
order	Phasmida	Phasmids, stick bugs
order	Psocodea	Psocids, Barklice, Booklice
order	Thysanoptera	Thrips

## Temporal coverage

**Data range:** 2025-9-02 – 2025-9-18; 2025-9-10 – 2025-10-11; 2026-5-05 – 2026-5-13.

### Notes

For the foliage beatings and AAS, 34 samplings were carried out during September 2025, between the 2nd and the 18th and 36 samplings were carried out during May 2026, between 5 and 15 May.

The 12 SLAM traps were set up between 10 September and 11 October 2025.

## Usage licence

### Usage licence

Creative Commons Public Domain Waiver (CC-Zero)

## Data resources

### Data package title

Arthropod assemblages in EU-certified organic orchards and surrounding hedgerows on Terceira Island (Azores, Portugal).

### Resource link

https://doi.org/10.15468/unbsgv

### Alternative identifiers

https://www.gbif.org/dataset/af6973c1-2f73-4464-ab2b-c2975b66cd88

### Number of data sets

2

### Data set 1.

#### Data set name

Event Table

#### Data format

Darwin Core Archive

#### Character set

UTF-8

#### Download URL

https://ipt.gbif.pt/ipt/resource?r=orchards_ter_biol

#### Data format version

1.3

#### Description

The dataset was published in the Global Biodiversity Information Facility platform, GBIF (Wallon et al. 2026). The following data-table includes all the records for which a taxonomic identification of the species was possible. The dataset submitted to GBIF is structured as a sample event dataset that has been published as a Darwin Core Archive (DwCA), which is a standardised format for sharing biodiversity data as a set of one or more data tables. The core data file contains 82 records (eventID).

**Data set 1.** 

Column label	Column description
eventID	Identifier of the events, unique for the dataset.
locationID	Identifier of the locations, unique for the dataset.
eventRemarks	Comments or notes about the dwc:Event with information about day vs. night sampling.
habitat	The habitat for an Event.
fieldNumber	Unique identifier of each trap used in the field for sampling.
samplingProtocol	The methods or protocols used during an Event.
sampleSizeValue	A numeric value for a measurement of the size (time duration, length, area or volume) of a sample in a sampling Event.
sampleSizeUnit	The unit of measurement of the size (time duration, length, area or volume) of a sample in a sampling Event.
samplingEffort	The amount of effort expended during an Event.
eventDate	The date-time or interval during which an Event occurred.
year	Year of the event.
month	Month of the event.
day	Day of the event.
continent	The name of the continent on which the Location occurs (Azores Archipelago).
islandGroup	The name of the island group in which the Location occurs (Azores Archipelago).
island	The name of the island on which the Location occurs (Terceira).
country	The full, unabbreviated name of the next smaller administrative region than stateProvince (county, shire, Department etc.) in which the Location occurs (Portugal).
countryCode	The standard code for the country in which the Location occurs (PT).
municipality	The full, unabbreviated name of the next smaller administrative region than county (city, municipality etc.) in which the Location occurs.
locality	The specific description of the place.
locationRemarks	Comments or notes about the Location.
minimumElevationInMetres	The lower limit of the range of elevation (altitude, usually above sea level), in metres.
decimalLatitude	Approximate centre point decimal latitude of the field site in GPS coordinates.
decimalLongitude	Approximate centre point decimal longitude of the field site in GPS coordinates.
geodeticDatum	Standard Global Positioning System coordinate reference for the location of the sample collection points.
coordinateUncertaintyInMetres	Uncertain value of coordinate metrics.
coordinatePrecision	Value in decimal degrees to a precision of six decimal places.
georeferenceSources	Navigation system used to record the location of sample collections.
datasetName	Name of the dataset.

### Data set 2.

#### Data set name

Occurrence table

#### Data format

Darwin Core Archive

#### Character set

UTF-8

#### Download URL

https://ipt.gbif.pt/ipt/resource?r=orchards_ter_biol

#### Data format version

1.3

#### Description

The dataset was published in the Global Biodiversity Information Facility platform, GBIF (Wallon et al. 2026). The following data table includes all the records for which a taxonomic identification of the species was possible. The dataset submitted to GBIF is structured as an occurrence table that has been published as a Darwin Core Archive (DwCA), which is a standardised format for sharing biodiversity data as a set of one or more data tables. The core data file contains 1,863 records (occurrenceID).

**Data set 2.** 

Column label	Column description
eventID	Identifier of the events, unique for the dataset.
type	The type of the related resource.
licence	Reference to the licence under which the record is published.
institutionID	An identifier for the institution publishing the data.
collectionID	An identifier for the collection or dataset from which the record was derived.
collectionCode	The acronym identifying the collection or dataset from which the record was derived.
institutionCode	The code of the institution publishing the data.
datasetName	Name of the dataset.
basisOfRecord	The nature of the data record.
recordedBy	A list (concatenated and separated) of names of people, groups or organisations who performed the sampling in the field.
occurrenceID	Identifier of the record, coded as a global unique identifier.
occurrenceStatus	A statement about the presence or absence of a Taxon at a Location
organismQuantity	A number or enumeration value for the quantity of Organisms.
organismQuantityType	The type of quantification system used for the quantity of Organisms.
sex	The sex of the biological individual(s) represented in the occurrence.
lifeStage	The age class or life stage of the Organism(s) at the time the Occurrence was recorded.
establishmentMeans	Statement about whether a Organism has been introduced to a given place and time through the direct or indirect activity of modern humans. We used the controlled vocabulary: endemic, native, introduced, uncertain.
identifiedBy	A list of names of people, groups or organisations who assigned the Taxon to the subject.
dateIdentified	The date on which the subject was determined as representing the Taxon.
scientificName	The full scientific name, with authorship and date information if known.
kingdom	Kingdom name.
phylum	Phylum name.
class	Class name.
order	Order name.
family	Family name.
genus	Genus name.
specificEpithet	Specific epithet name.
infraspecificEpithet	Name of the lowest or terminal infraspecific epithet of the scientific name.
taxonRank	Lowest taxonomic rank of the record.
scientificNameAuthorship	The authorship information for the scientificName formatted according to the conventions of the applicable nomenclaturalCode.
identificationRemarks	Dalberto Teixeira Pombo (DTP) collection's morphospecies number attributed to specimens identified.

## Additional information

A total of 15,739 specimens (adults and juveniles) were collected. A total of 299 morphospecies were generated, of which 196 were identified to species or subspecies level and assigned a valid scientific name. These species or subspecies represent 13,854 specimens. Of the remaining 103 morphospecies, 12 were identified to genus level, 85 to family level and six at order level only. Endemic taxa were poorly represented, with 11 taxa and 285 specimens, whereas native taxa dominated the assemblage, comprising 61 morphospeciess and 8,223 specimens. Exotic taxa were represented by 189 morphospecies (n = 6,836 specimens) and, finally, 38 morphospecies remain of uncertain colonisation status (n = 395 specimens).

Spiders, which are amongst the most important generalist predators in orchard ecosystems and can contribute substantially to natural pest control (Michalko et al. 2019), were well represented, with 50 species and 3,465 specimens. Amongst Coleoptera, several families played an important beneficial role, particularly Coccinellidae, which were well represented with 237 specimens and act as major aphid and scale predators during both larval and adult stages (Obrycki and Kring 1998, Hodek 2012) and Staphylinidae, with over 22 species and 149 specimens, many of which are active predators in both soil and canopy environments (Bohac 1999). Additional beetle families, such as Cryptophagidae, Latridiidae, Mycetophagidae, Nitidulidae and Silvanidae, contributed mainly through decomposition processes and indirect ecosystem regulation (Crowson 1981). Neuroptera were also present, albeit in lower abundance (33 specimens), with lacewing larvae acting as effective predators of aphids, mites, thrips and scale insects (New 2001). Within Hemiptera, predatory subgroups were recorded, including Anthocoridae (74 specimens), which are important predators of thrips, mites and aphids and a single specimen of Nabidae, another generalist predatory group (Lattin 1999). Dermaptera were also present (40 specimens) and likely contributed to nocturnal predation on aphids and other small orchard pests (Rankin and Palmer 2009).

Finally, a notable feature of the dataset is the high abundance of ants, represented by 4,325 specimens. Most of these individuals (4,194 specimens) were identified as *Lasius grandis* Forel, 1909, making it the dominant species in terms of abundance. This predominance likely reflects the suitability of orchard habitats for ants, owing to the diversity of available food resources, including floral products and honeydew produced by sap-feeding insects, such as aphids and scale insects. *Lasius grandis* is a common and widespread species in Mediterranean and Macaronesian agroecosystems, where it thrives in disturbed habitats and forms large colonies (Wetterer et al. 2006, Zina et al. 2020).

The dataset includes four species that are new records for Terceira Island: *Dipoena melanogaster* (C.L. Koch, 1837), *Textrix pinicola* Simon, 1875, *Stenocaecilius caboverdensis* (Meinander, 1966) and *Euconnus campestris campestris* (L.W. Schaufuss, 1866).

*Dipoena melanogaster* (C.L. Koch, 1837), which is represented by a single juvenile specimen, is a small theridiid spider commonly associated with warm and dry habitats, where it typically lives in vegetation and preys on other small arthropods, particularly ants and other spiders. It has been previously recorded on the Archipelago, on São Miguel Island.

*Textrix pinicola* Simon, 1875, a spider from the Agelenidae family, is represented by three juveniles specimens and was previously observed in the Archipelago, on Flores Island. It is a funnel-weaving species typically associated with dry, open to semi-wooded habitats and is an active nocturnal predator feeding on small arthropods that become trapped in its web.

*Stenocaecilius caboverdensis* (Meinander, 1966), which is represented by two adult specimens, is a psocid species usually associated with vegetation and tree bark, where it feeds on microflora, such as algae, fungi and lichens growing on plant surfaces. It has been previously recorded in the Archipelago, on the islands of Santa Maria and São Miguel.

Finally, *Euconnus campestris campestris* (L.W. Schaufuss, 1866), a beetle from the Staphylinidae family (Scydmaeninae), is represented by three adult specimens and was previously recorded in the Azores from São Miguel and Faial Islands. It typically inhabits leaf litter, moss and decaying organic matter in woodland and other humid habitats, where it preys on small soil invertebrates, particularly mites.

These records contribute to improving the current knowledge of the arthropod fauna of the Island and highlight the importance of continued biodiversity surveys in the Azores.

Although these newly-recorded species are not currently recognised as direct biological control agents, the presence of predatory taxa amongst them contributes to a better understanding of the arthropod food web associated with Azorean orchards. Such biodiversity inventories provide an important baseline for future studies assessing ecosystem services, including the natural regulation of pest populations.

Overall, this dataset provides a standardised baseline for comparing arthropod assemblages between orchard interiors and hedgerows in Azorean organic fruit-production systems.

## Figures and Tables

**Table 1.** Geographic coordinates of the six organic orchard systems sampled on Terceira Island (Azores, Portugal). Coordinates are reported in decimal degrees (WGS84).

**Site**	**decimalLatitude**	**decimalLongitude**
Fontinhas	38.746111	-27.103889
São Pedro	38.664167	-27.244444
São Mateus	38.657778	-27.254444
São Bartolomeu	38.674722	-27.291944
Terra-Chã	38.685833	-27.268611
Conceição	38.675	-27.214722
